# Response to electroconvulsive therapy in treatment-resistant depression: nationwide observational follow-up study

**DOI:** 10.1192/bjo.2023.5

**Published:** 2023-02-14

**Authors:** Adam Nygren, Johan Reutfors, Lena Brandt, Robert Bodén, Axel Nordenskjöld, Mikael Tiger

**Affiliations:** Centre for Pharmacoepidemiology, Division of Clinical Epidemiology, Department of Medicine, Solna, Karolinska Institutet, Sweden; Department of Medical Sciences, Psychiatry, Uppsala University, Sweden; University Health Care Research Centre, Faculty of Medicine and Health, Örebro University, Sweden; Centre for Psychiatry Research, Department of Clinical Neuroscience, Karolinska Institutet & Stockholm Health Care Services, Region Stockholm, Sweden

**Keywords:** Depressive disorders, electroconvulsive therapy, epidemiology, outcome studies, antidepressants

## Abstract

**Background:**

Previous studies have not investigated response rates after electroconvulsive therapy (ECT) in patients with non-psychotic treatment-resistant depression (TRD).

**Aims:**

To assess and compare the response rate of ECT for patients with TRD and non-TRD, in a large and clinically representative patient sample.

**Method:**

Patients aged ≥18 years, who were treated for a unipolar, non-psychotic depressive episode with at least one ECT session as part of a first-time, index ECT series between 1 January 2011 and 31 December 2017 were included from the Swedish National Quality Register for ECT. Patients who had initiated a third consecutive trial of antidepressants or add-on medications before start of ECT were classified as having TRD. Patients not meeting criteria for TRD were classified as non-TRD. The main outcome was response to ECT according to the Clinical Global Impressions – Improvement Scale (CGI-I), scored as 1 or 2 (‘very much’ or ‘much improved’ after ECT, respectively). Logistic regression was used to compare outcome measures between TRD and non-TRD, adjusting for potential confounders.

**Results:**

A total of 4244 patients were included. Of these, 1121 patients had TRD and 3123 patients had non-TRD. The CGI-I response rate was 65.9% in the TRD group compared with 75.9% in the non-TRD group (adjusted odds ratio 0.64, 95% CI 0.54–0.75). Older age and more severe depression were predictors of response in patients with TRD.

**Conclusions:**

A clear majority of patients with TRD, as well as patients with non-TRD, responded to ECT, although the response rate was somewhat lower for TRD.

Patients with depression who do not respond well to initial treatment trials are commonly referred to as having treatment-resistant depression (TRD). Although definitions of treatment resistance have varied over time, in several recent studies it has been defined as failure to achieve remission with two or more adequate antidepressant treatment trials.^1,2^ TRD is associated with increased mortality, loss of function and lower quality of life, as well as high societal costs.^2,3^

Electroconvulsive therapy (ECT) is an effective treatment for depression^4^ and can be considered for patients with TRD, as reflected in guidelines from several countries.^5,6^ In patients with psychotic depression, ECT is often considered a first-line treatment regardless of treatment resistance.^6^ Although some observational studies have reported a significantly worse ECT outcome in patients with treatment resistance,^7–9^ others have not.^10–16^ Some of these studies were small and patients were treated mainly with tricyclic antidepresssants,^8,16^ differing from today's practice in which selective serotonin reuptake inhibitors are more commonly used. In two meta-analyses, history of medication failure was predictive of a lower response rate after ECT,^17,18^ with one study reporting response rates of 58% and 70%^18^ and the other reporting remission rates of 48% and 65%^17^ for patients with and without previous failure of an adequate antidepressant trial, respectively. In the included studies, treatment resistance was defined by at least one failed antidepressant trial of adequate dose and duration, not using today's more common definition of two failed antidepressant trials. Moreover, many studies included patients with psychotic depression. Thus, previous studies have not addressed the particularly relevant clinical question of response rates after ECT in non-psychotic, unipolar depression resistant to multiple previous pharmacological treatment attempts. The aim of the present study was to assess and compare the response rate of ECT for non-psychotic, unipolar TRD (defined by at least two failed antidepressant trials of adequate dose and duration), in relation to the response rate among patients with non-TRD, in a nationwide patient sample.

## Method

### Data sources

The Swedish National Quality Register for ECT (Q-ECT) has had national coverage since 2011, and around 90% of all patients treated with ECT in Sweden accept inclusion onto the register. Data are registered after completed treatment, separately for index and continuation series, and include information on patient characteristics, severity of symptoms, indications for therapy, electrical stimulus parameters and seizure characteristics, and scores on the Clinical Global Impressions^19^ – Severity (CGI-S; before and after treatment) and Improvement (CGI-I; after treatment) scales. For the CGI-S and CGI-I, a clinician compares the patient's condition after treatment to that of before treatment, according to a seven-point scale ranging from 1 (very much improved) to 7 (very much worse). Evaluations of ECT effect are usually performed the day after, or at most a week after, the final session in the treatment series. The National Patient Register (NPR) contains patient data including main and secondary diagnoses and procedures according to the International Statistical Classification of Diseases and Related Health Problems (ICD), with revision 10 used since 1997, as well as dates for in-patient episodes. It has near complete coverage of both in-patient and out-patient specialised care, with out-patient care gradually included since 2001. The Prescribed Drug Register (PDR) contains data on all dispensed prescribed drugs in Sweden since July 2005. Drugs that are administered in hospitals are not included. The Swedish Longitudinal Integration Database for Health Insurance and Labour Market Studies (LISA) is held by Statistics Sweden and includes integrated demographic data.

### Study population

**Fig. 1:**
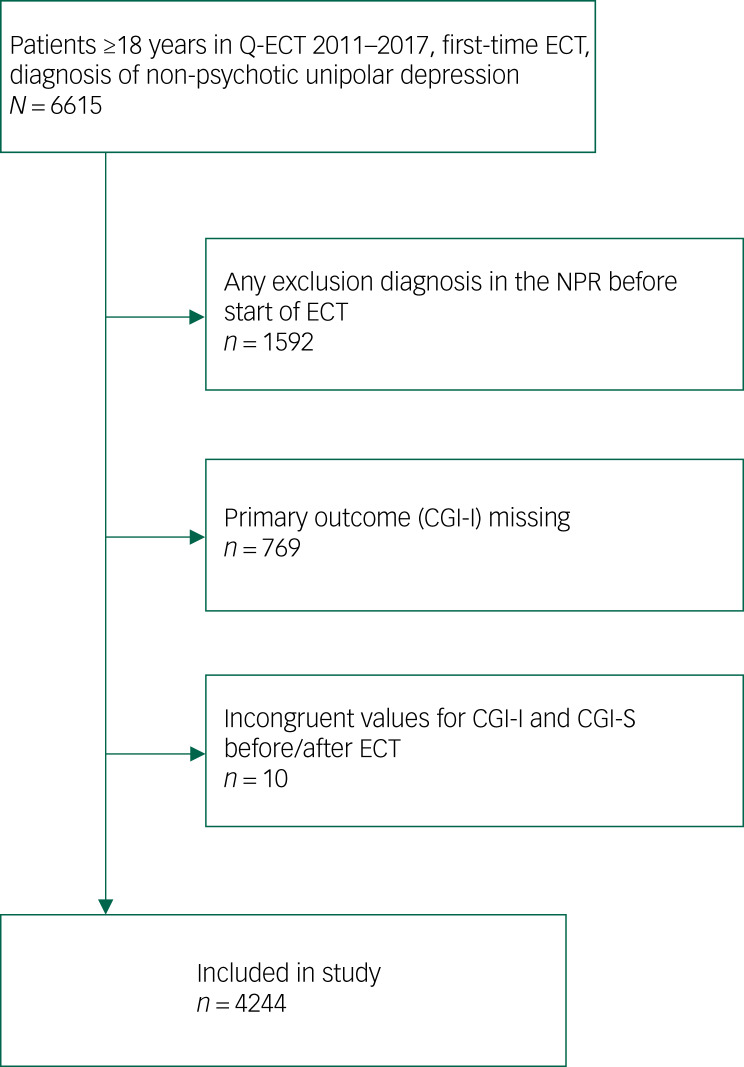
Flow chart of study inclusion. CGI-I, Clinical Global Impressions – Improvement Scale; CGI-S, Clinical Global Impressions – Severity Scale; ECT, electroconvulsive therapy; Q-ECT Swedish National Quality Register for ECT; NPR, Swedish National Patient Register.

From the Q-ECT, we identified patients who were aged ≥18 years and had been treated for a unipolar, non-psychotic depressive episode (ICD-10^20^ codes of F32 for depressive episode or F33 for recurrent depression, excluding F32.3 and F33.3 for psychotic depression) with at least one ECT session as part of a first-time, index ECT series in Sweden between 1 January 2011 and 31 December 2017 (Fig. 1). When registration of treatment indication was lacking or unclear in the Q-ECT, diagnostic information was added from the NPR. This resulted in 6615 eligible patients with a diagnosis of depression treated with ECT. Patients with any registered lifetime diagnosis of bipolar disorder, manic or hypomanic episode, psychotic disorder or dementia were excluded, as were patients lacking data on the primary outcome measure (CGI-I score). After these exclusions, a total of 4244 patients were included in the final analyses.

### Exposure

Patients were classified as having TRD if at least two subsequent pharmacological treatment trials for depression (a different Anatomic Therapeutic Chemical classification code, or an antidepressant add-on medication) were recorded in the PDR during the 2 years before ECT. To be considered adequate, a treatment trial needed to be at least 28 days long, reflecting a duration commonly required for antidepressants to have effect. The duration of each filled prescription was estimated from package size, dosage and prescription text. Add-on medications were defined as lithium, risperidone, olanzapine, aripiprazole and quetiapine, which are recommended for the treatment of TRD by guidelines.^5^ Patients not meeting criteria for TRD were classified as ‘non-TRD’. A validation study^21^ has shown that patients identified as TRD with this register-based method have similar characteristics as adaptations of clinical methods. In patients with depression, incidence rates of TRD as identified by register-based methods are similar in Swedish^2^ and Danish^22^ studies.

Additionally, we categorised exposure according to the number of pharmacological treatment trials before ECT, ranging from zero to five or more.

### Covariates

Income, education level and marital status were collected from Statistics Sweden. Any lifetime diagnosis of personality disorder (ICD-10 codes F60–F61), anxiety disorder (ICD-10 codes F40–F42) or substance use disorder (ICD-10 codes F10–F16, F18 and F19) was noted from the NPR, as were previous admissions for depression (ICD-10 codes F32–F33). Treatment setting was collected from the Q-ECT except when unavailable, in which case NPR data was used. Use of benzodiazepines, pregabalin and other anti-epileptics (except clonazepam and pregabalin) at the time of ECT was assessed with data on prescription fills from the PDR. Technical parameters of ECT were attained from the Q-ECT.

### Outcome measures

Outcome variables were retrieved from the Q-ECT. The main outcome measure was response to ECT, defined as CGI-I scores of 1–2 (very much improved or much improved). The secondary outcome measure was remission, defined as patients who were assessed as very much improved (CGI-I score of 1).

### Statistical analysis

Social and clinical characteristics, as well as technical parameters of administered ECT, were categorised and tabulated. Subsequently, patients with TRD were compared with patients with non-TRD by *χ*^2^-test. *P*-values of <0.05 were considered statistically significant. Further, outcome measures for the two groups were compared using logistic regression to calculate crude and adjusted odds ratios, together with their corresponding 95% confidence intervals and *P*-values, for response in patients with TRD compared with non-TRD. The following covariates were included simultaneously in the adjusted model, selected because of their relevance as potential confounders based on the literature: age, gender, anxiety disorder, personality disorder, substance use disorder and depression severity (CGI-S score before treatment). We also explored the impact of all of the available independent variables. Covariates were added one at a time to a base model consisting of age and gender. None of the covariates changed the estimated odds ratio by >10% for TRD versus non-TRD. Subsequently, unadjusted and adjusted odds ratios of ECT response were calculated stratified by TRD status for each of the variables included in the model, comparing each category to a reference category. We further compared ECT response for exposure categories of zero to five or more antidepressant trials in the same 4244 patients, using the adjusted model.

### Preregistration

The methodology and statistical approach for this study were preregistered on the Open Science Framework (available at https://osf.io/ay6bk).

### Ethical approval

The authors assert that all procedures contributing to this work comply with the ethical standards of the relevant national and institutional committees on human experimentation and with the Helsinki Declaration of 1975, as revised in 2008. All procedures involving human patients were approved by the Regional Ethical Vetting Board in Uppsala (approval number 2014/174). Individual informed consent was not required because this was a register-based study of anonymised data.

## Results

### Study population

Out of the 4244 included patients, 1121 patients were categorised as having TRD based on previous prescription fills in the PDR, with the remaining 3123 patients categorised as having non-TRD. As presented in Table 1, the proportion of women was significantly higher in patients with TRD. Patients with TRD were more often married, whereas differences in age, education level and income were non-significant. Higher proportions of patients with TRD had anxiety disorder (64.8% *v*. 42.9%), personality disorder (11.6% *v*. 7.4%) and substance misuse disorder (23.4% *v*. 17.9%). An out-patient ECT treatment setting was more common in patients with TRD (16.3% *v*. 6.5%). There were also significantly more previous hospital admissions for depression in the TRD group, as well as recent prescription fills of benzodiazepines and anti-epileptics. The majority of patients with TRD or non-TRD received six to ten sessions of ECT (64.5% *v*. 67.4%, respectively; see Table 1), but a higher proportion of patients with TRD received more than ten sessions (19.2% *v*. 15.9%; *P* = 0.01). In the first session, no significant difference between groups was found for electrode placement, whereas seizure length was slightly shorter in patients with TRD.

**Table 1:** Sociodemographic and clinical characteristics of patients with versus without treatment-resistant depression at start of electroconvulsive therapy, and parameters of administered electroconvulsive therapy

	TRD, *n =* 1121		Non-TRD, *n =* 3123		*χ*^2^^a^
	*n*	%	*n*	%	*P*-value
Gender					<0.001
Male	435	38.8	1426	45.7	
Female	686	61.2	1697	54.3	
Age, years					0.20
≤29	176	15.7	562	18.0	
30–49	335	29.9	953	30.5	
50–64	286	25.5	784	25.1	
≥65	324	28.9	824	26.4	
Married					0.003
Yes	461	41.1	1127	36.1	
No	658	58.7	1987	63.6	
Missing	2	0.2	9	0.3	
Education					0.26
≤9 years	242	21.5	743	23.5	
10–12 years	304	27.0	821	26.0	
≥13 years	576	51.2	1568	49.7	
Missing	4	0.4	24	0.8	
Income^b^					0.05
Low	490	43.7	1287	41.2	
Middle	593	52.9	1678	53.7	
High	36	3.2	149	4.8	
Missing	2	0.2	9	0.3	
Psychiatric comorbidity					
Anxiety disorder	727	64.8	1355	42.9	<0.001
Personality disorder	132	11.6	235	7.4	<0.001
Substance use disorder	265	23.4	565	17.9	<0.001
Treatment setting					<0.001
In-patient	936	83.5	2909	93.1	
Out-patient	183	16.3	203	6.5	
Missing	2	0.2	11	0.4	
Previous admission for depression					<0.001
None	505	45.0	1826	58.5	
1–3	503	44.9	1089	34.9	
4–9	100	8.9	189	6.1	
10–28	13	1.2	19	0.6	
Other psychotropic use^c^					
Benzodiazepines	677	60.4	1299	41.6	<0.001
Anti-epileptics except pregabalin	116	10.3	161	5.2	<0.001
Pregabalin	104	9.3	121	3.9	<0.001
Depression severity (CGI-S)					<0.001^d^
Borderline	4	0.4	6	0.2	
Mild	43	3.8	65	2.1	
Moderate	275	24.5	640	20.5	
Marked	569	50.8	1530	49.0	
Severe	186	16.6	723	23.2	
Among the most extreme	6	0.5	39	1.2	
Not assessed/not available	38	3.4	120	3.8	
Number of ECT sessions					0.04
1–5	183	16.3	519	16.6	
6–10	723	64.5	2106	67.4	
11–44	215	19.2	498	15.9	
Electrode placement					0.09^e^
Unilateral	1078	96.2	2959	94.7	
Bitemporal	20	1.8	109	3.5	
Bifrontal	17	1.5	36	1.2	
Other	2	0.2	2	0.1	
Missing	4	0.4	17	0.5	
Charge, mA					0.46
<250	276	24.6	751	24.0	
250–499	477	42.6	1185	37.9	
≥500	125	11.2	347	11.1	
Missing	243	21.7	840	26.9	
Pulse width, ms					0.1
<0.50	224	20.0	523	16.7	
0.50–1.00	648	57.8	1760	56.4	
Missing	249	22.2	840	26.9	
Seizure length, s					0.01
<30	163	14.5	349	11.2	
30–59	486	43.4	1259	40.3	
≥60	193	17.2	598	19.1	
Missing	279	24.9	917	29.4	

TRD, treatment-resistant depression; CGI-S, Clinical Global Impression – Severity Scale; ECT, electroconvulsive therapy; mA, milliampere; ms, millisecond.

a. Missing excluded from analysis. *P*-values refer to a null hypothesis that all categories of the variable are equal.

b. Low- and high-income groups defined as bottom and top 20 percentiles of income.

c. Prescription fills within 90 days before ECT.

d. CGI-S scores of 2–3 analysed as one group.

e. Comparing unilateral to remaining placements.

### Outcomes

Patients with TRD had a lower CGI-I response rate compared with patients with non-TRD (65.9% *v*. 75.9%), corresponding to an adjusted odds ratio of 0.64 (95% CI 0.54–0.75) (Table 2). The CGI-I remission rate was 16.4% for TRD and 25.7% for non-TRD (adjusted odds ratio 0.64, 95% CI 0.53–0.77).

**Table 2:** Electroconvulsive therapy response in patients with versus without treatment-resistant depression

	TRD, *n* = 1121	Non-TRD, *n* = 3123	Crude odds ratio		Adjusted odds ratio	
	Response, *n* (%)	Response, *n* (%)	Odds ratio (95% CI)	*P*-value	Odds ratio (95% CI)	*P*-value
Primary outcome: CGI-I response	739 (65.9)	2369 (75.9)	0.62 (0.53–0.71)	<0.001	0.65 (0.55–0.76)	<0.001
Secondary outcome: CGI-I remission	184 (16.4)	804 (25.7)	0.57 (0.47–0.68)	<0.001	0.64 (0.53–0.77)	<0.001

TRD, treatment-resistant depression; CGI-I, Clinical Global Impressions – Improvement Scale.

When response rates were examined separately by stratified covariate categories (Table 3), they were found to increase by age, ranging from 47.7% (≤29 years) to 75.6% (≥65 years) in patients with TRD, and from 58.2% (≤29 years) to 85.3% (≥65 years) in patients with non-TRD, corresponding to adjusted odds ratios between the oldest and youngest patients of 3.74 (95% CI 2.47–5.68) in TRD and 3.74 (95% CI 2.88–4.86) in non-TRD. There was no significant difference in response related to anxiety disorder or personality disorder in patients with TRD, whereas in patients with non-TRD, response rates were lower among patients with either of these diagnoses. Stratifying for depression severity, response rates were significantly higher in severely ill patients than in those who were markedly ill, with an adjusted odds ratio of 1.49 (95% CI 1.02–2.19) in patients with TRD and 1.40 (95% CI 1.11–1.76) in patients with non-TRD. Remission was similarly higher with increasing age and more severe depression in both groups, whereas other covariates had no significant effect on remission in patients with TRD (Supplementary Table 1 available at https://doi.org/10.1192/bjo.2023.5).

**Table 3:** Electroconvulsive therapy response in patients with versus without treatment-resistant depression, by covariate categories

	TRD, *n* = 1121					Non-TRD, *n* = 3123				
	Response (%)	Crude odds ratio (95% CI)	*P*-value^a^	Adjusted odds ratio (95% CI)	*P*-value^a^	Response (%)	Crude odds ratio (95% CI)	*P*-value^a^	Adjusted odds ratio (95% CI)	*P*-value^a^
Gender			0.38		0.66			0.11		0.37
Male	280 (64.4)	Reference: 1		Reference: 1		1101 (77.2)	Reference: 1		Reference: 1	
Female	459 (66.9)	0.89 (0.69–1.15)		0.94 (0.72–1.23)		1268 (74.7)	1.15 (0.97–1.35)		1.08 (0.91–1.29)	
Age, years			<0.001		<0.001			<0.001		<0.001
≤29	84 (47.7)	Reference: 1		Reference: 1		327 (58.2)	Reference: 1		Reference: 1	
30–49	218 (65.1)	2.04 (1.41–2.96)		2.25 (1.53–3.31)		689 (72.3)	1.88 (1.51–2.34)		1.87 (1.49–2.34)	
50–64	192 (67.1)	2.24 (1.52–3.29)		2.54 (1.69–3.82)		650 (82.9)	3.49 (2.71–4.48)		3.27 (2.53–4.22)	
≥65	245 (75.6)	3.40 (2.30–5.02)		3.74 (2.47–5.68)		703 (85.3)	4.18 (3.23–5.39)		3.74 (2.88–4.86)	
Anxiety disorder			0.92		0.53			<0.001		0.001
No	259 (65.7)	Reference: 1		Reference: 1		1426 (80.0)	Reference: 1		Reference: 1	
Yes	480 (66.0)	1.01 (0.78–1.31)		1.09 (0.83–1.44)		943 (70.4)	0.60 (0.50–0.70)		0.74 (0.62–0.89)	
Personality disorder			0.02		0.56			<0.001		<0.001
No	664 (67.1)	Reference: 1		Reference: 1		2239 (77.5)	Reference: 1		Reference: 1	
Yes	75 (56.8)	0.64 (0.45–0.93)		0.89 (0.59–1.34)		130 (55.6)	0.36 (0.28–0.48)		0.55 (0.41–0.75)	
Substance use disorder			0.20		0.65			<0.001		0.13
No	573 (66.9)	Reference: 1		Reference: 1		1983 (77.5)	Reference: 1		Reference: 1	
Yes	166 (62.6)	0.83 (0.62–1.10)		0.93 (0.69–1.26)		386 (68.6)	0.63 (0.52–0.78)		0.84 (0.67–1.05)	
Depression severity (CGI-S score before ECT)			<0.001		<0.001			<0.001		<0.001
Missing^b^	27 (71.1)	1.12 (0.54–2.30)		1.19 (0.56–2.49)		88 (73.3)	0.79 (0.52–1.21)		0.80 (0.51–1.24)	
Borderline to moderate	178 (55.3)	0.56 (0.42–0.75)		0.54 (0.40–0.72)		462 (65)	0.53 (0.44–0.65)		0.57 (0.46–0.70)	
Marked	391 (68.7)	Reference: 1		Reference: 1		1188 (77.6)	Reference: 1		Reference: 1	
Severe to extreme	143 (74.5)	1.33 (0.92–1.92)		1.49 (1.02–2.19)		631 (82.8)	1.39 (1.11–1.73)		1.40 (1.11–1.76)	

TRD, treatment-resistant depression; CGI-I, Clinical Global Impressions – Severity Scale; ECT, electroconvulsive therapy.

a. *P*-values refer to a null hypothesis that all categories of the variable are equal.

b. Missing included as a separate category.

Response rates were 74.6% of 1976 patients receiving benzodiazepines, 63.5% of 277 patients receiving anti-epileptics and 63.6% of 225 patients receiving pregabalin, compared with 73.2% of all 4244 patients. Adding benzodiazepine use to the base model did not change the odds ratio for response, whereas anti-epileptics and pregabalin use decreased the odds ratios by 1.6% and 2.3%, respectively.

Comparing outcomes across different numbers of antidepressant treatment trials before ECT (Supplementary Table 2) in the same 4244 patients, we found that response rates gradually declined from 82.1% in patients with no previous treatment trial, to 63.4% in those with five or more treatment trials before ECT; remission rates declined from 34.8% in previously untreated patients, to 13.4% after five or more treatment trials. Adjusted odds ratios were 0.43 (95% CI 0.29–0.64) for response and 0.35 (95% CI 0.22–0.55) for remission in patients with five or more trials compared with those with no previous treatment trials.

## Discussion

### Main findings

In this large, nationwide study of patients treated with ECT for unipolar, non-psychotic depression, we found high response rates in both groups, with 65.9% of patients with TRD assessed as much or very much improved after treatment, compared with 75.9% of patients with non-TRD. Lower proportions of patients had remission defined as the highest possible improvement assessment after treatment, with 16.4% compared with 25.7% for TRD and non-TRD, respectively. The differences in response and remission remained significantly lower in TRD even after statistical adjustment for a number of potential confounders. Nearly all of the difference in response rates could be attributed to the lower remission in patients with TRD, as evident from the 10% difference in response rates juxtaposed with the 9.3% difference in remission rates. We identified age and depression severity as factors associated with higher response rates in both patients with TRD and non-TRD, whereas anxiety or personality disorders were only associated with odds of response in patients with non-TRD.

### Comparison with previous findings

Our study is the first to primarily investigate resistance to multiple medication trials as a predictor of ECT outcome, as well as the largest sample yet to assess the association between any treatment resistance and ECT outcome. Response and remission were defined by CGI-I score after ECT, whereas most previous studies used definitions of response or remission based on depression rating scales.^17,18^ Despite this, response rates after ECT were similar to a meta-analysis including a total of 1175 patients from 11 studies, finding response in 58% of patients with resistance to at least one medication compared with 70% in patients without medication resistance before ECT.^18^

Although lower than for non-TRD, our finding of an ECT response rate of 65.9% in patients with TRD is high when compared with the Sequenced Treatment Alternatives to Relieve Depression (STAR*D) study, where treatment steps 3 and 4 – consisting of either switching to another class of antidepressant or augmentation treatments with lithium or T_3_ after two previous failed treatment trials – each prompted response in <17% of patients.^23^ Given that patients in our study were selected for ECT, more often were in-patients (in contrast to the out-patient setting of STAR*D) and likely had more severe depressive symptoms with higher likelihood of ECT response, our results may not necessarily be inferable to patients with less severe depression. To our knowledge, no studies have been conducted directly comparing antidepressant treatment and ECT for TRD.

Although we are not aware of any previous study stratifying ECT response by the number of previous treatment trials before ECT, the gradually declining response and remission rates by each increase in the number of trials found here is in line with observations made in earlier studies,^7,8^ where ECT response rates were lower with higher levels of previous antidepressant trial potency; however, results from a smaller study^10^ did not show a similar trend.

Various mechanisms that might explain previous findings from studies^10–16^ where response rates were not lower in patients with treatment resistance are plausible. First, previous studies may have been underpowered. In line with that explanation, two meta-analyses of predictors of ECT outcome^17,18^ showed significant differences comparing patients with and those without treatment resistance. The largest of these included a total of 1175 patients compared with the 4244 included in this study.^18^ Second, some studies that did not find a lower response in patients with treatment resistance used bilateral ECT,^12,14,15^ which might explain the more similar response rates in patients with and those without treatment resistance. Finally, several studies included patients with psychotic features, unlike ours, which may be of relevance since such features have been found to predict a better ECT response, with a meta-analysis finding response rates of 78% in psychotic depression compared with 71% in non-psychotic depression.^24^ Response rates in patients with TRD and non-TRD may accordingly become more similar when this condition is included, and with psychotic depression there is a risk of ‘pseudo-TRD’, since an adequate previous pharmacological treatment of psychotic depression has been shown to be rare among patients treated with ECT.^25^

In previous studies on predictors of ECT response, higher age and depression severity, as well as absence of anxiety and personality disorders, have been found to be significantly associated with a better ECT effect in depression,^18,26^ but this has not been studied specifically among patients with TRD. In the present study, we demonstrated that patients with TRD did not have significantly higher ECT response or remission depending on anxiety or personality disorders, whereas patients with non-TRD did. Older age and higher depression severity, however, were significantly associated with better odds of treatment response and remission in both patients with TRD and non-TRD.

The proportion of patients with TRD in this study (26.4%, 1121 out of 4244) was lower than patients with medication resistance in previous studies of patients treated with ECT. This may partly be because of the stricter requirement of two previous trials used in the study, and partly because of differences in treatment guidelines.

### Limitations

Diagnoses in the registers used for this study were recorded in regular clinical settings, meaning that diagnostic methods may have varied, although the validity of psychiatric diagnoses has been found to be high in both the NPR^27^ and Q-ECT.^28^ The outcome was measured with the CGI-I instead of depression rating scales, and assessments were non-blinded. The method used for defining TRD does not include non-pharmacological treatments, does not require switching between antidepressant classes and makes assumptions about medication failure when a new prescription is filled, meaning that some patients may have been misclassified as having TRD if they did not actually take the collected medication or dropped out because of side-effects. Related to this, because the PDR does not include data on in-hospital medication, there is a possibility that some patients who had TRD when treated as in-patients were misclassified in our study as having non-TRD. However, data from the Q-ECT show that the mean length of stay before starting ECT is 4 days (with an average total length of stay of 29 days for patients treated with ECT), and that only 5% of patients start ECT after more than 27 days of in-patient care (enabling 28-day trials of antidepressants). Add-on medications such as quetiapine may sometimes have been prescribed for other indications than for treatment of depression, such as for anxiety or sleep problems. Also, although only the previous 2 years of antidepressant fills were considered, the method did not exclude antidepressant use for preceding depressive episodes or indications other than depression. Further, although patients with any previous registration of ECT were excluded, there may have been cases where patients had previous ECT that was not registered in the Q-ECT or NPR, but who were selected for treatment because of previous response, likely increasing chances of a positive outcome. Such patients would likely be treated with ECT early and not go on to develop TRD, and may thus have contributed somewhat to the higher response rates in non-TRD. Moreover, data on the technical ECT parameters were limited to the first session, meaning any change in electrode placement or stimulus charge during the treatment course would not be detected. Because charge titration is not used in Sweden, we were also not able to provide data on stimulus charge exceeding the seizure threshold. Finally, our study does not address duration of treatment effects. A recent Swedish trial comparing ECT to racemic ketamine infusions for unipolar depression showed that after remission achieved by ECT, around 64% of patients relapsed within 12 months, with most of these being in the first 6 months.^29^ There are previous results to support a higher rate of relapse in patients with preceding treatment resistance than in those without.^30^

### Interpretation and conclusion

The association between TRD and a lower ECT response may represent a true effect of TRD on positive ECT outcome, such as if patients with TRD have a subtype of depression that is inherently more difficult to treat across treatment modalities. However, another possibility is that there is residual unmeasured confounding, which could contribute to the association. First, because of limitations in the available data in the Q-ECT, patients were already treated with ECT at study inclusion and may thus have been selected as suitable for ECT based on unmeasured clinical factors. Patients who were treated with ECT before they had developed TRD may have had characteristics possibly associated with improved ECT effect, such as rapidly worsening symptoms or a clear episodic character of depression, whereas for patients with TRD, a perceived lack of options may have resulted in them being given ECT despite possible negative factors such as a less clear-cut diagnosis of depression. Second, the rate of personality disorder in this material was relatively low compared with other studies, such as a European multicentre study^31^ where personality disorder was present in 50.5% of patients with TRD and 37.1% of patients with non-TRD. If there were more undiagnosed patients with personality disorder in the TRD group in our study, this could explain the lower response in patients with TRD to some extent, given the lower response rates we found in patients with non-TRD and diagnosed personality disorder. Although the possible mechanisms above cannot be excluded, TRD status should be seen as a prognostic clinical marker associated with somewhat lower ECT response.

In conclusion, results of this study of real-world data on unipolar, non-psychotic depression indicate that a clear majority of patients with depression respond to ECT. Response rates to ECT were found to be somewhat lower in the TRD group than in the non-TRD group, but were still encouraging relative to available alternatives in TRD, such as further antidepressant trials. In patients with TRD, as well as patients with non-TRD, older age and depression severity were corroborated as ECT response markers.

## Data Availability

The data used for this study may not be shared by the authors to a third party, according to the ethical permission granted for its use. It is accessible by application to the Swedish authorities (The National Board of Health and Welfare and Statistics Sweden).

## Supplementary material

For supplementary material accompanying this paper visit http://doi.org/10.1192/bjo.2023.5. click here to view supplementary material

## References

[1] Conway CR, George MS, Sackeim HA. Toward an evidence-based, operational definition of treatment-resistant depression: when enough is enough. JAMA Psychiatry 2017; 74(1): 9–10. 27784055 10.1001/jamapsychiatry.2016.2586

[2] Reutfors J, Andersson TM, Brenner P, Brandt L, DiBernardo A, Li G, Mortality in treatment-resistant unipolar depression: a register-based cohort study in Sweden. J Affect Disord 2018; 238: 674–9. 29966932 10.1016/j.jad.2018.06.030

[3] Johnston KM, Powell LC, Anderson IM, Szabo S, Cline S. The burden of treatment-resistant depression: a systematic review of the economic and quality of life literature. J Affect Disord 2019; 242: 195–210. 30195173 10.1016/j.jad.2018.06.045

[4] UK ECT Review Group. Efficacy and safety of electroconvulsive therapy in depressive disorders: a systematic review and meta-analysis. Lancet 2003; 361(9360): 799–808. 12642045 10.1016/S0140-6736(03)12705-5

[5] National Institute of Health and Care Excellence (NICE). Depression in Adults: Treatment and Management (Update). NICE, 2022. 35977056

[6] Milev RV, Giacobbe P, Kennedy SH, Blumberger DM, Daskalakis ZJ, Downar J, Canadian Network for Mood and Anxiety Treatments (CANMAT) 2016 clinical guidelines for the management of adults with major depressive disorder: section 4. Neurostimulation treatments. Can J Psychiatry 2016; 61(9): 561–75. 27486154 10.1177/0706743716660033 PMC4994792

[7] Prudic J, Haskett RF, Mulsant B, Malone KM, Pettinati HM, Stephens S, Resistance to antidepressant medications and short-term clinical response to ECT. Am J Psychiatry 1996; 153(8): 985–92. 8678194 10.1176/ajp.153.8.985

[8] Prudic J, Sackeim HA, Devanand DP. Medication resistance and clinical response to electroconvulsive therapy. Psychiatry Res 1990; 31(3): 287–96. 1970656 10.1016/0165-1781(90)90098-p

[9] Dombrovski AY, Mulsant BH, Haskett RF, Prudic J, Begley AE, Sackeim HA. Predictors of remission after electroconvulsive therapy in unipolar major depression. J Clin Psychiatry 2005; 66(8): 1043–9. 16086621 10.4088/jcp.v66n0813

[10] Pluijms EM, Birkenhager TK, Huijbrechts IP, Moleman P. Influence of resistance to antidepressant pharmacotherapy on short-term response to electroconvulsive therapy. J Affect Disord 2002; 69(1–3): 93–9. 12103456 10.1016/s0165-0327(00)00378-5

[11] van den Broek WW, de Lely A, Mulder PG, Birkenhager TK, Bruijn JA. Effect of antidepressant medication resistance on short-term response to electroconvulsive therapy. J Clin Psychopharmacol 2004; 24(4): 400–3. 15232331 10.1097/01.jcp.0000130551.70878.56

[12] Husain SS, Kevan IM, Linnell R, Scott AI. Electroconvulsive therapy in depressive illness that has not responded to drug treatment. J Affect Disord 2004; 83(2–3): 121–6. 15555704 10.1016/j.jad.2004.05.006

[13] Kho KH, Zwinderman AH, Blansjaar BA. Predictors for the efficacy of electroconvulsive therapy: chart review of a naturalistic study. J Clin Psychiatry 2005; 66(7): 894–9. 16013905 10.4088/jcp.v66n0712

[14] de Vreede IM, Burger H, van Vliet IM. Prediction of response to ECT with routinely collected data in major depression. J Affect Disord 2005; 86(2–3): 323–7. 15935255 10.1016/j.jad.2005.03.008

[15] Rasmussen KG, Mueller M, Knapp RG, Husain MM, Rummans TA, Sampson SM, Antidepressant medication treatment failure does not predict lower remission with ECT for major depressive disorder: a report from the consortium for research in electroconvulsive therapy. J Clin Psychiatry 2007; 68(11): 1701–6. 18052563 10.4088/jcp.v68n1109

[16] Heijnen WT, van den Broek WW, Birkenhager TK. Treatment failure with a tricyclic antidepressant followed by lithium addition and response to subsequent electroconvulsive therapy. J Clin Psychiatry 2008; 69(12): 1887–91. 19014754 10.4088/jcp.v69n1206

[17] Heijnen WT, Birkenhager TK, Wierdsma AI, van den Broek WW. Antidepressant pharmacotherapy failure and response to subsequent electroconvulsive therapy: a meta-analysis. J Clin Psychopharmacol 2010; 30(5): 616–9. 20814336 10.1097/JCP.0b013e3181ee0f5f

[18] Haq AU, Sitzmann AF, Goldman ML, Maixner DF, Mickey BJ. Response of depression to electroconvulsive therapy: a meta-analysis of clinical predictors. J Clin Psychiatry 2015; 76(10): 1374–84. 26528644 10.4088/JCP.14r09528

[19] Guy W. *ECDEU Assessment Manual for Psychopharmacology.* US Department Of Health, Education, and Welfare, Public Health Service, Alcohol, Drug Abuse, and Mental Health Administration, National Institute of Mental Health, Psychopharmacology Research Branch and Division of Extramural Research Programs, 1976.

[20] World Health Organization (WHO). The ICD-10 Classification of Mental and Behavioural Disorders. WHO, 1993.

[21] Hägg D, Brenner P, Reutfors J, Li G, DiBernardo A, Bodén R, A register-based approach to identifying treatment-resistant depression-comparison with clinical definitions. PLoS One 2020; 15(7): e0236434. 32730324 10.1371/journal.pone.0236434 PMC7392234

[22] Gronemann FH, Jorgensen MB, Nordentoft M, Andersen PK, Osler M. Incidence of, risk factors for, and changes over time in treatment-resistant depression in Denmark: a register-based cohort study. J Clin Psychiatry 2018; 79(4): 17m11845. 10.4088/JCP.17m11845 29873959

[23] Rush AJ, Trivedi MH, Wisniewski SR, Nierenberg AA, Stewart JW, Warden D, Acute and longer-term outcomes in depressed outpatients requiring one or several treatment steps: a STAR*D report. Ame J Psychiatry 2006; 163(11): 1905–17. 10.1176/ajp.2006.163.11.1905 17074942

[24] van Diermen L, van den Ameele S, Kamperman AM, Sabbe BCG, Vermeulen T, Schrijvers D, Prediction of electroconvulsive therapy response and remission in major depression: meta-analysis. Br J Psychiatry 2018; 212(2): 71–80. 29436330 10.1192/bjp.2017.28

[25] Mulsant BH, Haskett RF, Prudic J, Thase ME, Malone KM, Mann JJ, Low use of neuroleptic drugs in the treatment of psychotic major depression. Am J Psychiatry 1997; 154(4): 559–61. 9090348 10.1176/ajp.154.4.559

[26] Steinholtz L, Reutfors J, Brandt L, Nordanskog P, Thornblom E, Persson J, Response rate and subjective memory after electroconvulsive therapy in depressive disorders with psychiatric comorbidity. J Affect Disord 2021; 292: 276–83. 34134026 10.1016/j.jad.2021.05.078

[27] Ludvigsson JF, Andersson E, Ekbom A, Feychting M, Kim JL, Reuterwall C, External review and validation of the Swedish National Inpatient Register. BMC Public Health 2011; 11: 450. 21658213 10.1186/1471-2458-11-450 PMC3142234

[28] Ahmad I, Sandberg M, Brus O, Ekman CJ, Hammar Å, Landén M, Validity of diagnoses, treatment dates, and rating scales in the Swedish National Quality Register for Electroconvulsive Therapy. Nord J Psychiatry 2022; 76(2): 96–103. 34346852 10.1080/08039488.2021.1939416

[29] Ekstrand J, Fattah C, Persson M, Cheng T, Nordanskog P, Åkeson J, Racemic ketamine as an alternative to electroconvulsive therapy for unipolar depression: a randomized, open-label, non-inferiority trial (KetECT). Int J Neuropsychopharmacol 2022; 25(5): 339–49. 35020871 10.1093/ijnp/pyab088 PMC9154276

[30] Sackeim HA, Prudic J, Devanand DP, Nobler MS, Lisanby SH, Peyser S, A prospective, randomized, double-blind comparison of bilateral and right unilateral electroconvulsive therapy at different stimulus intensities. Arch Gen Psychiatry 2000; 57(5): 425–34. 10807482 10.1001/archpsyc.57.5.425

[31] Souery D, Oswald P, Massat I, Bailer U, Bollen J, Demyttenaere K, Clinical factors associated with treatment resistance in major depressive disorder: results from a European multicenter study. J Clin Psychiatry 2007; 68(7): 1062–70. 17685743 10.4088/jcp.v68n0713

