# Supplementary material for: Response to electroconvulsive therapy in treatment-resistant depression: nationwide observational follow-up study
Source: BJPsych Open. 2023 Feb 14;9(2):e35. doi: 10.1192/bjo.2023.5 (PMC9970162; doi:10.1192/bjo.2023.5)
Supplement: Supplementary file 1 [file bjosup.zip › S2056472423000054sup001.docx]

| **Supplementary table 1. ECT remission in TRD and non-TRD by covariate categories** | | | | | | | | | | |
| --- | --- | --- | --- | --- | --- | --- | --- | --- | --- | --- |
|  | **TRD, n=1,121** | | | | | **Non-TRD, n=3,123** | | | | |
|  | Remission (%) | Crude odds ratio (95% CI) | *P*-value^a^ | Adjusted odds ratio (95% CI) | *P*-value^a^ | Remission (%) | Crude odds ratio (95% CI) | *P*-value^a^ | Adjusted odds ratio (95% CI) | *P*-value^a^ |
| **Sex** |  |  | 0.79 |  | 0.49 |  |  | 0.18 |  | 0.39 |
| Males | 73 (20.2) | Ref=1 |  | Ref=1 |  | 362 (25.4) | Ref=1 |  | Ref=1 |  |
| Females | 111 (19.3) | 1.05 (0.76-1.44) |  | 1.13 (0.80-1.59) |  | 442 (26.0) | 0.97 (0.82-1.14) |  | 0.93 (0.78-1.10) |  |
| **Age (years)** |  |  | 0.001 |  | <0.001 |  |  | <0.001 |  | <0.001 |
| ≤29 | 15 (9.3) | Ref=1 |  | Ref=1 |  | 86 (15.3) | Ref=1 |  | Ref=1 |  |
| 30-49 | 52 (18.4) | 1.97 (1.08-3.62) |  | 2.25 (1.20-4.21) |  | 202 (21.2) | 1.49 (1.13-1.96) |  | 1.52 (1.15-2.03) |  |
| 50-64 | 45 (18.7) | 2.00 (1.08-3.72) |  | 2.33 (1.22-4.46) |  | 253 (32.3) | 2.64 (2.01-3.47) |  | 2.59 (1.95-3.43) |  |
| ≥65 | 72 (28.6) | 3.07 (1.70-5.53) |  | 3.71 (1.97-6.95) |  | 263 (31.9) | 2.60 (1.98-3.41) |  | 2.42 (1.82-3.20) |  |
| **Anxiety disorder** |  |  | 0.46 |  | 0.51 |  |  | <0.001 |  | 0.05 |
| No | 69 (21.2) | Ref=1 |  | Ref=1 |  | 515 (28.9) | Ref=1 |  | Ref=1 |  |
| Yes | 115 (18.8) | 0.89 (0.64-1.23) |  | 0.89 (0.63-1.26) |  | 289 (21.6) | 0.68 (0.57-0.8) |  | 0.84 (0.7-1.00) |  |
| **Personality disorder** |  |  | 0.10 |  | 0.69 |  |  | <0.001 |  | 0.02 |
| No | 169 (20.6) | Ref=1 |  | Ref=1 |  | 774 (26.8) | Ref=1 |  | Ref=1 |  |
| Yes | 15 (12.8) | 0.62 (0.35-1.09) |  | 0.88 (0.47-1.64) |  | 30 (12.8) | 0.40 (0.27-0.60) |  | 0.61 (0.41-0.93) |  |
| **Substance use disorder** |  |  | 0.92 |  | 0.35 |  |  | <0.001 |  | <0.001 |
| No | 140 (19.6) | Ref=1 |  | Ref=1 |  | 710 (27.7) | Ref=1 |  | Ref=1 |  |
| Yes | 44 (19.9) | 1.02 (0.7-1.48) |  | 1.21 (0.81-1.81) |  | 94 (16.7) | 0.52 (0.41-0.66) |  | 0.63 (0.49-0.81) |  |
| **Depression severity  (CGI-S before ECT)** |  |  | <0.001 |  | <0.001 |  |  | <0.001 |  | <0.001 |
| Missing^b^ | 7 (22.6) | 1.06 (0.45-2.47) |  | 1.08 (0.46-2.55) |  | 27 (22.5) | 0.93 (0.59-1.45) |  | 0.95 (0.60-1.49) |  |
| Borderline-Moderately ill | 22 (7.3) | 0.34 (0.21-0.56) |  | 0.33 (0.20-0.53) |  | 103 (14.5) | 0.54 (0.43-0.69) |  | 0.58 (0.45-0.74) |  |
| Markedly ill | 100 (21.3) | Ref=1 |  | Ref=1 |  | 365 (23.9) | Ref=1 |  | Ref=1 |  |
| Severely-Extremely ill | 55 (40.1) | 1.88 (1.29-2.75) |  | 2.09 (1.41-3.08) |  | 309 (40.6) | 2.18 (1.81-2.62) |  | 2.21 (1.83-2.67) |  |
| a. *P*-values refer to a null-hypothesis that all categories of the variable are equal. b. Missing included as a separate category.  ECT, electroconvulsive therapy; TRD treatment-resistant depression | | | | | | | | | | |
