# Supplementary material for: Response to electroconvulsive therapy in treatment-resistant depression: nationwide observational follow-up study
Source: BJPsych Open. 2023 Feb 14;9(2):e35. doi: 10.1192/bjo.2023.5 (PMC9970162; doi:10.1192/bjo.2023.5)
Supplement: Supplementary file 1 [file bjosup.zip › S2056472423000054sup002.docx]

| **Supplementary table 2. ECT response and remission for different numbers of previous treatment trials** | | | | | | | | | | |
| --- | --- | --- | --- | --- | --- | --- | --- | --- | --- | --- |
| Number of AD trials | Response (%) | Crude OR  (95% CI) | *P-*value^a^ | Adjusted OR  (95% CI) | *P-*value^a^ | Remission (%) | Crude OR  (95% CI) | *P-*value^a^ | Adjusted OR  (95% CI) | *P-*value^a^ |
| **0**  *n*=577 | 474 (82.1) | Ref=1 | <0.001 | Ref=1 | <0.001 | 201 (34.8) | Ref=1 | <0.001 | Ref=1 | <0.001 |
| **1**  *n*=1,369 | 1,039 (75.9) | 0.68  (0.54-0.88) |  | 0.78  (0.60-1.00) |  | 347 (25.3) | 0.64  (0.52-0.78) |  | 0.71  (0.57-0.88) |  |
| **2**  *n*=1,177 | 856 (72.7) | 0.58  (0.45-0.74) |  | 0.69  (0.53-0.90) |  | 256 (21.8) | 0.52  (0.42-0.65) |  | 0.61  (0.48-0.77) |  |
| **3**  *n*=618 | 419 (67.8) | 0.46  (0.35-0.60) |  | 0.55  (0.41-0.73) |  | 107 (17.3) | 0.39  (0.30-0.51) |  | 0.49  (0.37-0.65) |  |
| **4**  *n*=309 | 197 (63.8) | 0.38  (0.28-0.52) |  | 0.44  (0.31-0.61) |  | 51 (16.5) | 0.37  (0.26-0.52) |  | 0.45 (0.31-0.65) |  |
| **≥5**  *n*=194 | 123 (63.4) | 0.38  (0.26-0.54) |  | 0.43  (0.29-0.64) |  | 26 (13.4) | 0.29  (0.19-0.45) |  | 0.35  (0.22-0.55) |  |
| a. *P*-values refer to a null-hypothesis that all categories of the variable are equal.  ECT, electroconvulsive therapy; OR, odds ratio; AD, antidepressant | | | | | | | | | | |
